# Characteristics of Immune Checkpoint Inhibitors Trials Associated With Inclusion of Patients With HIV: A Systematic Review and Meta-analysis

**DOI:** 10.1001/jamanetworkopen.2019.14816

**Published:** 2019-11-08

**Authors:** Hadas Sorotsky, David Hogg, Eitan Amir, Daniel V. Araujo

**Affiliations:** 1 Department of Medical Oncology and Hematology, Princess Margaret Cancer Centre, Toronto, Ontario, Canada

## Abstract

This systematic review and meta-analysis examines factors associated with inclusion of patients with HIV in clinical trials of immune checkpoint inhibitors.

## Introduction

Since antiretroviral therapy became widely available in the late 1990s, there has been a dramatic reduction in AIDS-related deaths due to opportunistic infections.^1^ The life expectancy of people living with HIV (PLWH) receiving antiretroviral therapy now approaches that of the general population, particularly for those with a CD4^+^ T-cell count within the normal range.^2^ Conversely, cancer has become a leading cause of morbidity and mortality in PLWH. From 1995 to 2009, cancer resulted in nearly two-fold as many deaths per 100 000 person-years in PLWH than in the general population (327 deaths per 100 000 person-years vs 186 deaths per 100 000 person-years).^3^ Non–AIDS-defining cancers represent 70% of these deaths and will increase with aging of the PLWH population.^3^

During the past decade, clinical trials of immune checkpoint inhibitors (ICIs) have changed the treatment landscape of many cancers. Historically, PLWH have been excluded from participation in oncology trials, which may affect generalizability of findings. In this systematic review and meta-analysis, we investigated characteristics of ICI trials associated with inclusion of PLWH.

## Methods

We performed a systematic search using the key word “checkpoint” to identify prospective trials involving programmed cell death protein 1 (PD-1) and programmed death ligand 1 (PD-L1) or cytotoxic T-lymphocyte–associated protein 4 ICIs published in 6 high-profile general medicine and oncology journals (Table 1) between January 1, 2016 and March 31, 2019. Two independent reviewers (D.V.A. and H.S.) assessed articles, including supplements and protocols, as well as ClinicalTrials.gov data to determine whether PLWH were eligible for enrollment. In cases of ambiguity, results were discussed with a third reviewer (D.H.). This study followed the Preferred Reporting Items for Systematic Reviews and Meta-analyses (PRISMA) reporting guideline.

**Table 1.** Characteristics of Included Trials

Characteristic	Trials, No. (%) (N = 118)
Patients, No.	306 093
Mean (95% CI)	260.1 (206.8-313.5)
Median (interquartile range)	117.5 (51.5-403.5)
Journal	
* New England Journal of Medicine*	17 (14.4)
* Lancet*	10 (8.5)
* Lancet Oncology*	37 (31.4)
* Journal of Clinical Oncology*	37 (31.4)
* JAMA*	1 (0.8)
* JAMA Oncology*	12 (10.1)
* Annals of Oncology*	4 (3.4)
Publication date	
2016	27 (22.9)
2017	36 (30.5)
2018	38 (32.2)
2019	17 (14.4)
Involved PD-1 and PD-L1 ICIs	
No	10 (8.5)
Yes	108 (91.5)
Combination of ICI with other treatment	
No	74 (62.7)
Yes	44 (37.3)
Trial phase	
1	39 (33.1)
2	50 (42.4)
3	29 (24.6)
Multicenter	
No	8 (6.8)
Yes	110 (93.2)
Tumor type	
Lung	24 (20.3)
Melanoma	17 (14.4)
Urothelial	13 (11)
Renal cell carcinoma	8 (6.8)
Head and neck squamous cell carcinoma	7 (5.9)
Other	49 (41.5)
Line of treatment	
First line metastatic	40 (33.9)
Other	78 (66.1)
Patients with HIV allowed	
No	102 (95.3)
Yes	5 (4.7)
Missing	11
Funding	
Pharmaceutical industry	98 (83.1)
Academic	20 (16.9)

Abbreviations: ICI, immune checkpoint inhibitor; PD-1, programmed cell death 1; PD-L1, programmed-death ligand 1.

Logistic and linear regression were used to assess factors associated with inclusion of PLWH. We used the Peto odds ratio method for associations with 0-counts. *P* values were 2-tailed, and statistical significance was defined as *P* less than .05. No corrections were made for multiple significance testing. Data analysis was performed using SPSS statistical software version 25.0 (IBM) and Revmen review software version 5.3 (Cochrane) and conducted from June 1, 2019, to June 21, 2019.

## Results

Of 569 articles screened, 126 articles were trials of ICIs. We excluded 8 studies: 4 studies were duplicates, and 4 studies involved ICIs other than PD-1, PD-L1, or cytotoxic T-lymphocyte–associated protein 4. Our final analysis included 118 articles comprising 30 693 patients. Demographic data for the 118 articles are summarized in Table 1. For PLWH eligibility analysis, we excluded 11 trials owing to unavailability of data. Of 107 trials with PLWH inclusion criteria data, 5 trials (4.7%, 258 patients) allowed enrollment of PLWH. All 5 trials that allowed PLWH were academic, whereas no trials sponsored by a pharmaceutical company included PLWH (odds ratio, 259.77; 95% CI, 26.25-2570.61; *P* < .001) (Table 2). Only phase 2 trials permitted PLWH entry; however, this is likely a confounder, as most academic trials were phase 2 (17 of 20 trials; 85%). No other factors were associated with PLWH inclusion.

**Table 2.** Factors Associated With Inclusion of Patients with HIV in ICI Trials

Factor	Trials, No.			OR (95% CI)	*P* Value
	Patients With HIV Allowed		Total (N = 107)		
	No (n = 102)	Yes (n = 5)			
Funding					
Pharmaceutical industry	87	0	87	259.77 (26.25-2570.61)	<.001
Academic	15	5	20	1 [Reference]	
Publication date					
2016	24	0	24	1 [Reference]	NA
2017	29	1	30	0.16 (0.01-8.53)	.37
2018	34	3	37	0.18 (0.02-1.91)	.16
2019	15	1	16	0.08 (0.01-4.48)	.22
Combination of ICI with other treatment					
No	65	4	69	0.43 (0.04-4.07)	.47
Yes	37	1	38	1 [Reference]	
Involved PD-1 and PD-L1 ICIs					
No	8	1	9	0.34 (0.03-3.42)	.36
Yes	94	4	98	1 [Reference]	
Tumor type					
Lung	22	0	22	4.88 (0.58-40.74)	.14
Melanoma	15	0	15	4.15 (0.41-41.39)	.22
Urothelial	13	0	13	3.96 (0.36-43.14)	.26
Renal cell carcinoma	7	0	7	3.45 (0.18-63.66)	.40
Head and neck squamous cell carcinoma	6	1	7	0.58 (0.04-8.11)	.58
Other	39	4	43	1 [Reference]	NA
Line of treatment					
First line metastatic	35	2	37	0.78 (0.12-4.91)	.80
Other	67	3	70	1 [Reference]	
Trial phase					
1	36	0	36	6.93 (1.13-42.24)	.04
2	38	5	43	1 [Reference]	NA
3	28	0	28	5.76 (0.90-36.53)	.06

Abbreviations: ICI, immune checkpoint inhibitors; NA, not applicable; PD-1, programmed cell death 1; PD-L1, programmed-death ligand 1; OR, odds ratio.

## Discussion

Limitations of our study include the lack of multivariable adjustment and absence of reporting the justification for PLWH exclusion. Nonetheless, our results demonstrate an almost universal exclusion of PLWH from trials involving ICIs. There are no good justifications for this practice. A 2019 systematic review^4^ demonstrated that the safety profile of PD-1 or PD-L1 ICIs in PLWH was similar to that of the general population, with no unusual adverse events. A study by Uldrick et al^5^ of a phase 1 trial involving 30 patients with HIV and advanced cancer treated with pembrolizumab reported a safety profile in keeping with studies of participants without HIV. While academic studies were more likely to allow PLWH, even within this group only 5 trials permitted PLWH. Whereas the reasons for PLWH exclusion are not disclosed in any protocols, we suspect that this practice results from dogma rather than reasoned decision.

A recent American Society of Clinical Oncology task force^6^ recommended inclusion of PLWH in oncology trials, particularly patients with T-cell CD4^+^ counts of 350 cells/μL or higher, who constitute a group with intact immunological function and survival in keeping with the general population. In contrast, the exclusion of PLWH is unsupported by current data, denies patients the benefit of ICI therapy, and is ethically unjustifiable. We advocate for a broader inclusion of these patients in oncology trials.
